# A high-throughput assay for the comprehensive profiling of DNA ligase fidelity

**DOI:** 10.1093/nar/gkv898

**Published:** 2015-09-13

**Authors:** Gregory J. S. Lohman, Robert J. Bauer, Nicole M. Nichols, Laurie Mazzola, Joanna Bybee, Danielle Rivizzigno, Elizabeth Cantin, Thomas C. Evans

**Affiliations:** New England BioLabs, Inc., Ipswich, MA 01938-2723, USA

## Abstract

DNA ligases have broad application in molecular biology, from traditional cloning methods to modern synthetic biology and molecular diagnostics protocols. Ligation-based detection of polynucleotide sequences can be achieved by the ligation of probe oligonucleotides when annealed to a complementary target sequence. In order to achieve a high sensitivity and low background, the ligase must efficiently join correctly base-paired substrates, while discriminating against the ligation of substrates containing even one mismatched base pair. In the current study, we report the use of capillary electrophoresis to rapidly generate mismatch fidelity profiles that interrogate all 256 possible base-pair combinations at a ligation junction in a single experiment. Rapid screening of ligase fidelity in a 96-well plate format has allowed the study of ligase fidelity in unprecedented depth. As an example of this new method, herein we report the ligation fidelity of *Thermus thermophilus* DNA ligase at a range of temperatures, buffer pH and monovalent cation strength. This screen allows the selection of reaction conditions that maximize fidelity without sacrificing activity, while generating a profile of specific mismatches that ligate detectably under each set of conditions.

## INTRODUCTION

Polynucleic acid ligases, which join fragments of DNA and RNA, are cornerstone tools of modern molecular biology, fundamental to cloning, molecular diagnostics and high-throughput sequencing methodologies (1–7). DNA ligases catalyze the formation of a phosphodiester bond between 5′-phosphorylated DNA termini and the 3′-OH of DNA, with DNA ligases most frequently having the highest activity on DNA ‘nicks’ (breaks in the phosphodiester backbone of one strand of a dsDNA duplex having adjacent 3′-OH and 5′-phosphorylated termini) (3,4,6,7). DNA ligases are key components in Okazaki fragment processing, non-homologous end joining, and the sealing of spontaneous nicks and nicks left after base and nucleotide excision repair and thus indispensable for maintaining genome integrity (3–6).

The fidelity of mismatch ligation has been studied for a range of ligases (8), from the very promiscuous—e.g., T4 DNA ligase, able to ligate nearly all mismatches (8–13)—to highly discriminatory thermostable ligases—e.g., *Thermus thermophilus* (*Tth*) DNA ligase and *Pyrococcus furiosus* (*Pfu*) DNA ligase (14–19)—as well as a wide array of ligases of intermediate fidelity (for examples, see: (19–25)). The vast majority of characterized ligases discriminate much more stringently against mismatches on the side of the ligation junction providing the 3′-hydroxyl (the ‘upstream’ strand) than they do on the side of the junction providing the 5′-phosphorylated nucleoside (the ‘downstream’ strand) (8,9,14). In both positions, ligases generally discriminate more strongly against the large, helix-distorting purine:purine mispairs than pyrimidine:pyrimidine and purine:pyrimidine mispairs. Ligases also prefer helix-stabilizing, ‘wobble’ base pairs that can form multiple hydrogen bonds (such as G:T and A:G pairs) to those that form one or no hydrogen bonds. There are often exceptions to these general rules and the specific base-pair mismatches that can be ligated vary by enzyme: for example, *Tth* ligase will ligate the bulky pA:A base pairs but not pC:C. Further, a given enzyme does not always react the two permutations of a given base pair with equal efficiencies; for example, pT:G mispairs (pT in the active site, G in the complementary strand) are ligated much more efficiently by *Tth* ligase than pG:T mispairs (with pG in the active site, and T in the complement) (14). The mechanistic basis for fidelity is complex and the determining step clearly differs amongst studied enzymes, with evidence for discrimination both in DNA binding and in the various chemical steps, depending on the enzyme (8). While most ligase fidelity studies have been performed at low ionic strengths (< 25 mM Na^+^ or K^+^) where many ligases show the highest *in vitro* activity, it has also been found for several ligases (e.g., T4 DNA ligase and Human DNA ligases I and III) that increasing ionic strength leads to increased fidelity (8,9,26).

Thermostable ligases such as from *T. thermophilus* strain HB8 function at elevated temperature (45–75°C) and resist denaturing at very high temperatures (>95°C) (14,27–29). The ability of thermostable ligases to selectively join correctly base-paired annealed fragments with adjacent ends, as well as their ability to survive thermocycling, has led to their use in several ligation-based gene and single nucleotide polymorphism (SNP) detection techniques, including the ligase detection reaction (LDR), the ligase chain reaction (LCR) and padlock/molecular inversion probes (30–35). These techniques rely on the ligation of DNA probe sequences when annealed only to fully complementary target sequences and thus, DNA ligases that can discriminate between the ligation of correct and mismatched base pairs at the ligation junction. In LDR and LCR, successive rounds of melting, annealing and ligation are used to amplify the signal. Other related methods use only one round of ligation followed by detection of the ligated product through a variety of methods such as quantitative PCR (qPCR) or rolling circle amplification (for examples, see: (33,36–45)). Accurate detection of SNPs by these methods requires a very strong discrimination against mismatch ligation. Typical probes are designed to anneal to a unique region of the gene target, with the SNP to be interrogated at or very near to the ligation junction of the probe oligonucleotides. Ligation of the fully Watson–Crick (WC) base-paired probes must be very efficient to generate a strong positive signal, while ligation of probes mismatched at only a single base must be very inefficient in order to preserve a low background and allow the target of interest to be quantified in the presence of closely related sequences. Since the SNP sequences differ by only one nucleotide, the annealing temperature of the probe to the two sequences alone will not differ enough to prevent annealing and ligation to the sequence with one mismatch; thus, the ligase used must itself strongly discriminate against substrates containing base-pair mismatches.

Past studies on ligation fidelity have typically used sets of defined, nicked substrates, with one of the ligatable strands labeled with a radioactive moiety or a fluorophore. Substrates contained either fully WC base-paired sequences or introduced one or more mismatches at or near the ligation junction. Each substrate was then independently reacted with a ligase, products separated by gel electrophoresis, and either initial rates or final yields were used to generate profiles of ligase specificity (8,14,21,22). In this paper, we outline an extension of the past mismatch analysis techniques using detection of 3,6-fluorescein (FAM)-labeled ligation products by capillary gel electrophoresis (CE). As outlined in the companion manuscript to this paper, high throughput CE can allow for rapid and quantitative analysis of nucleic acid mixtures, including cases where the detected oligonucleotide products differ in size by as little as 1 or 2 nucleotides (46). Here, we couple the CE detection methodology with a highly multiplexed substrate pool design to allow the profiling of ligase mismatch fidelity in a rapid, parallel format. This method uses upstream and downstream probes of different lengths, keyed to the identity of the base at the ligation junction, leading to 16 FAM-labeled products of unique lengths. Separation and analysis via CE allows the simultaneous quantitation of the ligation of substrates covering all 256 possible base-pair combinations around the ligation junction (compared to only 32 in the most complex of prior studies) in one experiment (14). We have applied these mismatch ligation panels in the current study to profile the ligation fidelity of *Tth* DNA ligase in a high degree of detail under a range of conditions, varying reaction temperature, buffer pH and ionic strength. The method described herein allows for the rapid screening of ligases and buffer conditions for the optimization of LDR and related methods, and simple visualization of mismatch data permits quick analysis of mismatch ligation profiles and trends.

## MATERIALS AND METHODS

### General

Blunt/TA Ligase Master Mix, T4 DNA ligase, 10x T4 DNA ligase buffer, *T. thermophilus* strain HB8 DNA Ligase (sold commercially as *Taq* DNA Ligase for historical reasons), 9°N DNA ligase, 2 M KCl, 1 M MgCl_2_, 1 M DTT, 10 mM ATP and 50 mM NAD^+^ were obtained from New England BioLabs (NEB, Ipswich, MA). Tris-HCl (1 M) buffer stocks of pH 7.0, 7.5, 8.0, 8.5 and 9.0 (determined @ 25°C) were obtained from Amresco (Solon, OH). Triton X-100 (10%) was obtained from Sigma-Aldrich (St. Louis, MO). Ambion Nuclease-Free water was obtained from Life Technologies (Grand Island, NY). *Taq* DNA ligase reaction buffers (20 mM Tris-HCl, 25 mM KCl, 10 mM MgCl_2_,1 mM NAD^+^, 10 mM DTT, 0.1% Triton® X-100) were prepared as 10X stocks at pH 7.0, 7.5, 8.0, 8.5 and 9.0 @ 25°C. Oligonucleotide annealing buffer (10 mM Tris pH 7.5 @ 25°C, 50 mM KCl, 0.1 mM EDTA) was prepared as a 10X stock.

### Preparation of nicked dsDNA mismatch panels

**Figure 1.**
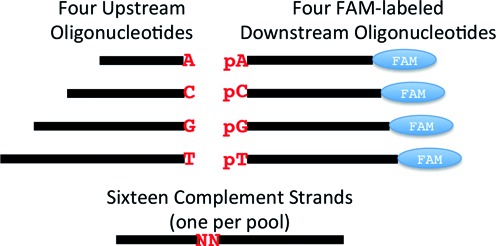
Schematic of multiplexed substrate pools. Each substrate pool contained a single splint with a defined NN at the ligation junction (e.g., AA, AC, AG…) along with all four upstream probes and all four FAM-labeled downstream probes. Each probe is of unique lengths that encode the base at the ligation junction: 20, 28, 36 and 44 bases for the 3′-A, C, G and T terminated upstream probes; 30, 32, 34 and 36 bases for the 5′-pA, pC, pG and pT terminated, 3′-FAM-labeled downstream probes. A total of 16 substrate pools were prepared, one for each unique splint.

HPLC purified, synthetic, single-stranded oligonucleotides were obtained from Integrated DNA Technologies (Coralville, IA) as lyophilized solids. The full sequences of the oligonucleotides for the nick mismatch panel can be found in Supplementary Figure S1. The panel (Figure 1) consists of four ‘upstream’ oligonucleotides that provide the 3′-OH at the ligation junction; four ‘downstream’ oligonucleotides that provide the 5′-phosphate at the ligation junction and are labeled with FAM at their 3′-terminus; and 16 complementary splint oligonucleotides. The upstream probes have an A, C, G or T at the ligation junction and are of lengths 20, 28, 36 and 44 bases, respectively. The downstream probes have a pA, pC, pG or pT at the ligation junction and are of lengths 30, 32, 34 and 36 bases, respectively. The two 3′ terminal bases in these sequences were TC for all four probes to minimize sequence effects on FAM fluorescence yield; probe lengths were increased by sequential addition of TC to the 3′ end (Supplementary Figure S1). The 16 splint oligonucleotides are all 50 bases in length, cover all combinations of bases (NN) at the ligation junction, and are otherwise of identical sequence complementary to the 20 upstream probe bases and 30 downstream probe bases closest to the ligation junction. The calculated melting temperature (*T*_m_) (IDT Oligo analyzer, http://www.idtdna.com/calc/analyzer) was ∼53°C for the 20 complementary bases upstream of the ligation junction and ∼71°C for the 30 complementary bases downstream of the ligation junction, calculated at 25 mM monovalent cation and 10 mM MgCl_2_. All oligonucleotides were dissolved in nuclease-free water to a final concentration of 100 μM, based on the quantitation provided by IDT.

The four downstream probes were combined into an equimolar mixture (25 μM each) and quantified by capillary electrophoresis using an Applied Biosystems 3730xl Genetic Analyzer (96 capillary array), as previously described (46,47) (Supplementary Figure S2A). The four upstream probes were likewise combined into an equimolar mixture (25 μM each) and quantified by reversed-phase HPLC performed on Agilent Liquid Chromatography/Mass Spectrometry Single Quad System 1200 Series (analytical) and Agilent 1200 Purification System, using an Agilent Eclipse Plus C18 column (3.0 × 60 mm, rapid resolution high density (RRHD) 1.8 micron particle size) at a flow rate of 0.5 ml/min, 60°C, with a binary gradient from solvent A (0.1 M triethylammonium acetate, pH 7) to solvent B (acetonitrile) (9% to 12.5% B over 20 min). Samples were monitored by UV-visible absorbance at 260 nm and the area of each peak normalized by the predicted extinction coefficient of each oligo to allow determination of the molar ratio (Supplementary Figure S2B).

Substrates were annealed in 1X oligonucleotide annealing buffer, combining one equivalent of one splint oligonucleotide with one equivalent each of the upstream and downstream probe mixtures (0.25 equivalents of each probe oligonucleotide relative to the splint oligonucleotide). The final concentrations were 10 μM splint, 2.5 μM each upstream probe, and 2.5 μM each downstream probe oligonucleotide. The mixtures were heated to 95°C for 2 min then cooled over 2 h to room temperature, then stored at −20°C. Thus, 16 substrate pools were prepared, each with a single splint, 4 upstream probes and 4 downstream probes. In each pool, there were consequently 16 total dsDNA nicked substrates, 1 perfectly WC base paired, 3 with one downstream mismatch but correctly base paired upstream of the ligation junction, 3 with one upstream mismatch but correctly base paired downstream of the ligation junction and 9 with mismatches on both sides of the ligation junction. The products of ligation of all 16 nicked substrates are of unique size and can be resolved, identified and quantified simultaneously by fluorescence-detected CE separation. Taking into account all 16 substrate pools with 16 dsDNA nicks each, all 256 possible base combinations at the ligation junction are represented in the panel.

### Mismatch ligation assay general protocol

Typical mismatch ligation protocols were run in a 96-well plate format. Each well contained one substrate pool (composed of one splint, four upstream probes and four downstream probes), with 16 wells total for each panel. Substrates were prepared as a 2X substrate concentration (400 nM total FAM label) in 1X reaction buffer, and 5 μl of the 2X substrates placed in each well. *Tth* ligase was likewise prepared as a 2X ligase concentration (4 nM ligase unless stated otherwise in the text) in 1X reaction buffer. Each plate containing the 2X substrate mixtures were first heated to 95°C for 1 min in a Bio-Rad T-100 Thermal Cycler with a heated top, then cooled to the ligation reaction temperature (55°C, unless stated otherwise). The 2X ligase mixture was added via eight-channel pipette, 5 μl per well and the plate sealed with Bio-Rad Microseal ‘B’ Seals. Plates were incubated 30 min at reaction temperature. At the end of the incubation the reaction was cooled to 10°C and quenched with 100 μl 5 mM EDTA per well. The quenched samples were then diluted further with ddH_2_O (10 μl sample in 100 μl water) then analyzed by capillary electrophoresis as described previously and in the accompanying manuscript (46,47). Positive control reactions were performed substituting 5 μl 2X Blunt/TA Ligase Master Mix (a high-activity formulation of T4 DNA ligase that readily ligates nicks containing multiple base-pair mismatches) for the *Tth* DNA ligase 2X preparation. The control reactions were incubated 24 h at 25°C, with quench and analysis by CE as above. CE analysis confirmed that all 16 possible ligation products could be observed in all substrate pools, with >85% of FAM-labeled probes competent for reaction to ligated DNA.

### CE data processing and analysis

**Figure 2.**
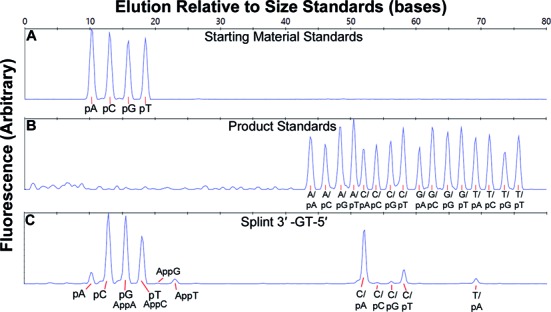
Sample CE traces, monitoring only the blue (FAM) channel. The Y-axis is arbitrary fluorescence; the X-axis indicates the elution time relative to the 120LIZ size standard (Applied Biosystems). (**A**) Elution of an equimolar mixture of the four starting oligonucleotide FAM-labeled downstream probes; from left to right the peaks are pA, pC, pG and pT. (**B**) Elution of a mixture of the 16 product standards; from left to right the peaks are A/pA, A/pC, A/pG, A/pT, C/pA, C/pC, C/pG, C/pT, G/pA, G/pC, G/pG, G/pT, T/pA, T/pC, T/pG, T/pT. (**C**) Elution of an experimental substrate pool (3′-GT-5′ splint) after ligation with 2 nM *Tth* DNA ligase for 30 min at 55°C in standard ligation buffer. Peaks were identified by comparison to standard, with clearly identifiable peaks for the C/pA, C/pT and T/pA ligation products. Peaks also appear at elution times consistent with the C/pC and C/pG products; these peaks are close to (C/pG) or below (C/pC) the minimum size that can be called confidently relative to baseline noise. Unreacted starting material peaks can also be identified, along with peaks resulting from 5′ adenylylation (AppN) of the starting probes in ligation reactions that failed to complete. AppG and AppT can be resolved, while AppA and AppC were found to coelute with pG and pT, respectively. The areas of all visible peaks were taken into account when calculating the yield of each product.

The output of the CE runs were analyzed using the Peak Scanner program (ABI, version 1.0). For the assay described here, only the ‘blue’ (FAM) channel was analyzed. Data for each run were recorded as the graphical plot of fluorescence versus elution time, typically represented as ‘base pairs’ relative to a co-eluting standard. Figure 2A shows the elution of the unligated starting pool of FAM-labeled downstream probes (from left to right, 5′-pA, pC, pG and pT terminated). Note that these compounds run smaller than true size relative to the standard set used in this assay. For small DNA fragments the elution time is increasingly dominated by the properties of the fluorophore; we have found FAM-labeled oligos to consistently run fast relative to the standard as shown, and this effect is more pronounced the shorter the oligonucleotide. Figure 2B shows synthetic standards (IDT) for the 16 possible ligation products. These products are identified by the terminal bases of the upstream/downstream probe pair they represent; e.g. A/pA is the oligonucleotide that corresponds to the 50 base ligation product of the 3′-A terminated upstream probe and 5′-pA terminated downstream probe, G/pT the 72 base product of ligation of the 3′-G terminated upstream probe and 5′-pT terminated downstream probe, etc. When discussing base pairs between the probes and the splint, the notation N:N or pN:N is used, where the base before the colon is the base in the probe and the base after the colon, the base in the complementary splint; e.g., C:G represents a C in the probe pairing with a G in the splint, and pG:A represents a G in the downstream probe pairing with an A in the splint. Figure 2C shows an example reaction of one substrate pool (four upstream probes, four downstream probes and one splint with 3′-GT-5′ at the ligation junction) reacted with 2 nM *Tth* DNA ligase for 30 min at 55°C. The consistency of the elution size relative to standard allows each ligation product to be positively identified in this complex mixture. In this experimental sample (Figure 2C), the major product identified matched the fully WC base-paired C/pA ligation product. Products resulting from the ligation of mismatched probes can also be seen, including the ligation products resulting from a correct upstream C:G base pair but mismatched downstream base pairs (the C/pC, C/pG and C/pT products resulting from ligation of nicks containing pC:T, pG:T and pT:T mispairs). Additionally, the ligation product resulting from a T:G upstream mismatch ligating to a correct pA:T base pair on the downstream side (the T/pA product) can be observed. Additional peaks can be observed eluting near the starting materials; based on previous work (47,48), we identified these as 5′-adenylylated (App) probe molecules resulting from aborted ligation reactions. The 5′-adenylylated probes AppG and AppT were resolved cleanly from the starting pN peaks, while AppA coelutes with pG and AppC coelutes with pT.

**Figure 3.**
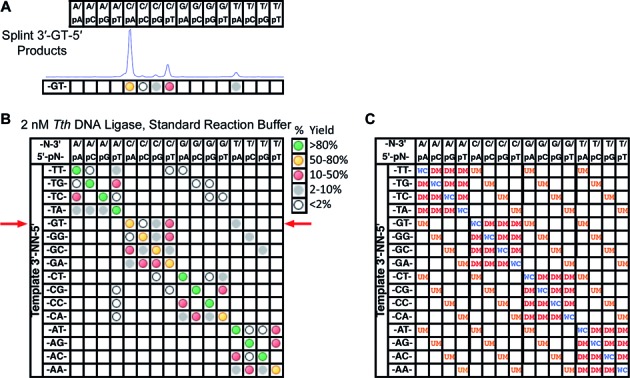
(**A**) Products formed upon incubation of the substrate pool containing the splint oligonucleotide with a 3′-GT-5′ at the ligation junction with 2 nM *Tth* DNA ligase in the standard reaction buffer (same data as Figure 2C). The CE trace was converted to a shorthand notation (below trace) marking the products observed as dots, where the color indicates the yield of that product. Assuming a maximum yield of one quarter of the total FAM fluorescence (the complete reaction of one downstream probe with one upstream probe), green represented >80% maximum yield, yellow 50–80%, red 10–50% and gray 2–10%. Open circles indicate product peaks were observed at <2%, a level generally not confidently distinguishable from baseline. (**B**) Mismatch ligation profile for the reaction of the complete panel with 2 nM *Tth* DNA ligase. Each reaction well contained 200 nM total FAM-labeled downstream probe oligonucleotides, 200 nM total unlabeled upstream probe oligonucleotides and 200 nM of one splint oligonucleotide (see Materials and Methods for the composition of an individual substrate pool). Reactions were incubated for 30 min at 55°C in the standard reaction buffer at pH 7.5. Each row represents a single ligation reaction including all four upstream probes and all four downstream probes and a single splint with the indicated bases at the ligation junction. Each column represents a single product of ligation identified by comparison with synthetic standards as described in Figure 2 and Materials and Methods. The red arrows indicate the row which corresponds to the reaction trace shown in Figure 3A. Experiments were performed in triplicate with the presented data the average of three runs. (**C**) Illustration of which products represent the ligation of fully base-paired or partially mismatched substrates. The main diagonal from top left to bottom right represents the ligation product that is the WC base-pairing partner of the splint present in the ligation pool (WC). The indicated cells represent ligation products resulting from fully base-paired upstream probes but a mismatch between the splint and the 5′-downstream probe terminus (DM), and ligation products resulting from fully base-paired downstream probes but a mismatch between the splint and the 3′-upstream probe terminus (UM). Unmarked squares represent positions mismatched at both sites flanking the ligation junction, which are rarely observed for *Tth* DNA ligase.

For each panel, all 16 substrate pools were run in parallel and the ligation products identified. Each peak was quantified as a percentage of total fluorescence based on peak area of all called peaks. The Peak Scanner program was used, as described in the accompanying manuscript (46), for peak picking, with a minimum peak height of 150 used to call peaks (just above background noise in a typical experiment). The results from a full ligation panel were visualized using a grid as shown in Figure 3. The bases listed on the left hand side of the grid (template 3′-NN-5′) identify the bases present in the splint oligonucleotide. Thus, each row represents one single CE trace. The columns represent one of the 16 possible ligation products identified by the upstream and downstream bases at the ligation junction. A dot is included in the cell if that product was identified in the CE trace. The color of the dot is used to encode the yield of each product, with green representing >80% maximum yield; yellow 50–80%; red 10–50%; and gray 2–10%. Open circles denote a detected peak <2% maximum yield (near the background of most experiments). Note that the maximum yield possible for any single product should be 25% of total fluorescence peak area, representing one of the four upstream probes reacting completely with one of the four downstream probes. Errors are assumed to be ±10% of the value determined from the peak size (i.e., maximum yields were actually 25% ± 2.5% of total fluorescence peak area). This error was estimated based on deviations from the expected 25% maximum value (typical observed yields in assay conditions resulting in high fidelity varied between 22% and 27% for the WC product) and the standard deviation observed across replicates. Error in peak area/yield determination arises from a variety of sources, including incomplete or variable annealing, difficulties inherent in precise oligonucleotide concentration determination, potential context dependent variations in FAM fluorescence and potential CE injection differences. These issues are discussed in more detail in the companion manuscript to this paper (46).

Figure 3A shows how the product region of the CE trace from Figure 2C was abstracted into the dot representation. The WC product is shown by the yellow dot below the C/pA column; the downstream mismatch products are shown in the open, gray and red circles under the C/pC, C/pG, and C/pT columns, and the upstream mismatch product is shown as the gray dot under the T/pA column. The qualitative visualization of the data in Figure 3B shows this abstraction for all 16 substrate pools (all 16 -NN- combinations in the splint). Assembly into the grid format allows the overall degree and type of mismatch ligation to be easily ascertained for all possible base-pair combinations. As illustrated in Figure 3C, the diagonal from top left to bottom right represents all fully base-paired (WC) ligation products; downstream mismatch (DM) ligation products are found around this primary axis, and upstream mismatch (UM) ligation products are found as off-diagonals.

### Initial velocity (V_0_) measurements for comparison of thermostable ligase activity

In order to compare the activity of DNA ligases across a range of buffer conditions, the initial velocity of ligation of a defined nicked dsDNA substrate was tested at 100 nM dsDNA and 250 pM *Tth* DNA ligase. The substrate used was a nicked dsDNA substrate composed of an upstream fragment of sequence (5′-GCG CAC CCT TAC CAC CAA GAC AGG ATC GTC CTT GC-3′), downstream fragment of sequence (5′-pTGA TCA TGC ATC GTT CCA CTG TGT CCG CGA CAT CTA CGT C-3′FAM) and complementary splint of sequence (5′-GAC GTA GAT GTC GCG GAC ACA GTG GAA CGA TGC ATG ATC AGC AAG GAC GAT CCT GTC TTG GTG GTA AGG GTG CGC-3′), with a predicted *T*_m_ of ∼76°C for both fragments. Initial rates were determined for a variety of reaction conditions. The effect of pH was measured in buffers over a range of Tris pH 7.0–9.0 (determined @ 25°C) at a reaction temperature of 55°C. The influence of temperature on rate was measured using the standard reaction buffer at pH 7.5 or 8.5 at a range of temperatures (45–75°C). The effect of salt on initial rate was measured using the reaction buffer pH 7.5 or 8.5 at a range of KCl concentrations (25–300 mM). Aliquots (2 μl) were quenched with stop solution (10 μl of 50 mM EDTA, 0.1% Triton X-100), diluted to a final FAM concentration of 0.5–1 nM and analyzed by CE as previously described (46,47). Time points were taken (typically at 20s, 30s, 1 min or 2 min intervals) such that the initial rate could be determined by a linear fit to the first 10–20% of reaction. Initial velocities reported are the average of three experiments with the error one standard deviation from the mean.

## RESULTS AND DISCUSSION

### The mismatch ligation profile of *Tth* DNA ligase under standard reaction conditions

The mismatch panel (see Materials and Methods for details on panel assembly and Supplementary Figure S1 for all sequences) was initially tested using the well-characterized *Tth* DNA ligase from strain HB8 (14). Reactions were performed at 2 nM DNA ligase, corresponding to the 1.6 U/μl concentration recommended by the manufacturer. The results of ligation of the mismatch panel at 55°C are shown in Figure 3B. Qualitatively, the results for this panel match the previously observed results from prior studies testing nicked substrates including mismatched base pairs in isolation (14). Mismatches were much better tolerated on the downstream side of the ligation junction than the upstream side, as expected for this ligase. The predominant upstream mismatches observed were the wobble base pairs G:T and T:G, as in the prior study, with T:G ligating more efficiently than G:T. On the downstream side of the ligation junction, in general pT:T, pT:G, pA:C and pC:A mismatches ligated quite readily, with lesser amounts of pG:T, pC:C, pA:A and pG:A mismatch ligation observed. In most cases at 2 nM ligase, little to no ligation from pG:G, pA:G, pC:C, pC:T and pT:C base pairs were observed. Again, these patterns match quite well the results of the study of mismatch nick ligation carried out previously (14). However, the current profile was generated rapidly in a single experiment and 30 min incubation, as compared to multiple long incubations of the ligase with each mismatch substrate in separate reactions as typically used in prior studies. A second reaction was run at 20 nM DNA ligase to better visualize low-frequency ligation events (data not shown). This experiment presented the same overall pattern of mismatch ligation products, but the yield of each mismatch was increased. Conversely, the yield of the WC product was decreased due to consumption of the probes in these increased mismatch ligations. Additionally small amounts of the ligation products resulting from A:C and C:A upstream mismatches and increased amounts of downstream side pC:C, pT:C and pC:T mismatches were observed, not previously identified as possible mismatches ligatable by *Tth* DNA ligase (14).

It was also interesting to note that while the overall pattern of mismatches ligated by *Tth* DNA ligase matches those previously reported, the use of this substrate panel shows that there are clear differences in the ability of the enzyme to ligate a particular mismatch depending on sequence context. For example, when the splint nucleosides were 3′-GN-5′ at the ligation junction, more mismatch ligation was observed than with the other splints. This corresponds to a C:G WC base pair between the upstream probe 3′-terminal base and the splint resulting in increased amounts of mismatch products on the downstream side of the ligation junction, as well as ligation of several downstream probe:splint base-pair mismatches (pG:T, pG:G, pC:C, pA:G, pC:T and pT:C base pairs) that were not observed for other upstream probe:splint base pairs. Thus, it appears that the identity of the correct base pair on one side of the ligation junction can significantly influence the mismatch ligation on the opposing side of the ligation junction in both degree and kind. Prior studies limited the substrate pool to those with only one of the four possible WC base pairs across the ligation junction from mismatches and thus, could not have observed this difference. For example, in the study of *Tth* ligase previously reported, when testing the ligation of mismatches on the downstream side of the ligation junction, only the T:A WC base pair was examined upstream of the junction (14).

For comparison of ligation by *Tth* DNA ligase to a low fidelity ligase, we have included the results of incubating the panel with 2 nM T4 DNA ligase for 30 min at 37°C in Supplementary Figure S3. Here we observed almost no discrimination against mismatches on the phosphorylated downstream probe side or against upstream T:T, T:G, G:T, C:A or A:C mismatches, though upstream probe A:G and G:A mismatches were not ligated. T4 DNA ligase could even measurably ligate some double mismatches involving G:T, T:G and T:T mispairs on both sides of the ligation junction.

### Effect of temperature on mismatch ligation

**Figure 4.**
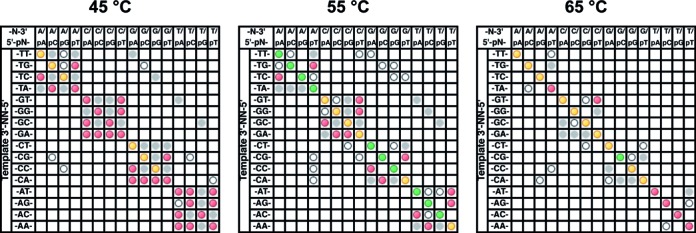
Mismatch ligation panel profiles for the reaction of the substrate panel with 2 nM *Tth* DNA ligase at 45°C, 55°C and 65°C. Reactions were incubated for 30 min at the stated reaction temperature in the standard reaction buffer at pH 7.5. Products were analyzed via CE and the data are displayed as described in the Materials and Methods section ‘CE Data Processing and Analysis’ and the caption of Figure 3.

The mismatch panel was tested with DNA ligase at three temperatures (45, 55 and 65°C; Figure 4). As expected, the fidelity rises as temperature increases, as evidenced by the sharp reduction in mismatch products observed and an increase in yield of the WC product. Total ligation activity appears to drop at 65°C, with reduced yields of many WC products compared to the reaction at 55°C. While the ligase is fully active at this temperature, 65°C is 10°C degrees above the predicted *T*_m_ for the upstream probe and very near the *T*_m_ of the downstream probe. Likely the low steady state concentration of fully annealed dsDNA substrate leads to restricted rates of product formation. However, good fidelity and high yields are achieved at 55°C, despite being above the predicted *T*_m_ of the upstream probe. Comparing the results at 55°C to the results at 45°C, the yield of the fully WC base-paired product is enhanced relative to the mismatch products, but the same overall pattern of mismatch ligation remains evident. Upstream of the ligation junction, largely only G:T and T:G mispairs are evident, while the major downstream side mismatches are pT:T, pT:G, pC:A and pA:C at both temperatures. The ligation of less-favored mismatches, such as pG:T, pG:G, pC:C, pC:T and pT:C, are more evident at 45°C, but greatly reduced or eliminated at 55°C. At 65°C the ligation of mismatches upstream of the ligation junction is completely eliminated, and all downstream mismatches are greatly reduced with only some products resulting from the ligation of pT:G, pT:T and pC:A mismatches detectable. Mismatch ligation is stronger for cases where the terminal base of the upstream probe is a correctly base-paired C:G or G:C, with the products of ligating downstream pA:C, pA:A and pG:A mismatches also detectable in this case, but not when the base pair upstream of the ligation junction is T:A or A:T.

The generalizability of this conclusion was checked using a second mismatch panel with much higher predicted *T*_m_ (∼75°C for both upstream and downstream probes), incubating at 65°C and 75°C (Supplementary Figure S4). The mismatch pattern at 75°C (above the *T*_m_ for both strands) for this high *T*_m_ substrate panel looks nearly identical to the pattern of the original panel at 55°C, while the high *T*_m_ panel at 65°C (∼10°C below the *T*_m_) shows higher degrees of mismatch ligation of most downstream mispairs and shows clear upstream mismatch peaks for G:T and T:G mispairs as well as trace A:C, C:A, C:T and T:T mispairs. These results indicate that it is ideal to run high-fidelity ligations 0–5°C above the *T*_m_ of the probe oligonucleotides (calculated for the buffer conditions used). Further, in any situation where many sets of probes are being simultaneously annealed and ligated to nucleic acid targets, probes with high *T*_m_ are very likely to show a much greater degree of off-target ligation than low *T*_m_ probes. Thus, to maximize fidelity of a multiplexed probe set, it would be desirable to match the *T*_m_ of all probes as closely as possible so that a single temperature optimal for all probes can be chosen for the assay (for examples of *T*_m­_ selection for previous optimized sets of multiplexed LDR probes, see (49–51)). This approach may require differential lengths for the annealing region to achieve proper *T*_m_ matching.

### Effect of pH on mismatch ligation

**Figure 5.**
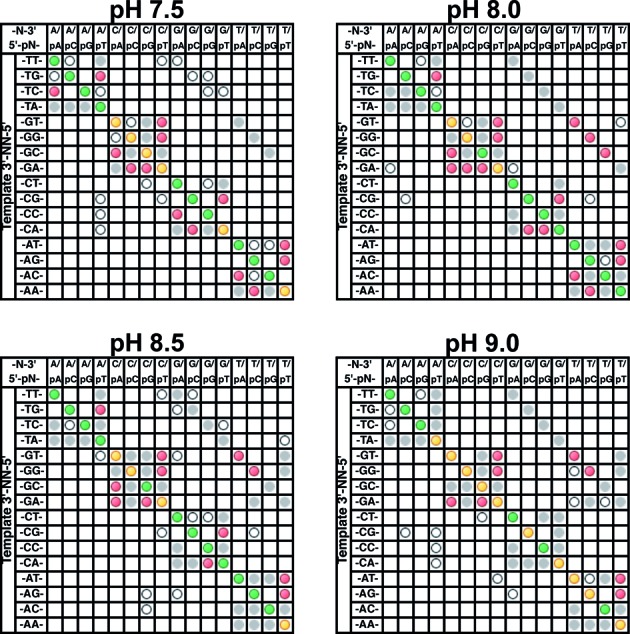
Mismatch ligation panel profiles for the reaction of the substrate panel with 2 nM *Tth* DNA ligase at buffer pH 7.5–9.0. Each reaction well contained 200 nM FAM-labeled oligonucleotides (see Materials and Methods for the composition of an individual substrate pool). Reactions were incubated for 30 min at 55°C in the standard reaction buffer at pH 7.5, 8.0, 8.5 or 9.0. The stated pH of the buffer stocks (Amresco) were determined at 25°C, not the reaction temperature. Products were analyzed via CE and the data are displayed as described in the Materials and Methods section ‘CE Data Processing and Analysis’ and the caption of Figure 3.

The effect of buffer pH on the mismatch ligation profile was tested at pH 7.5, 8.0, 8.5 and 9.0 (determined @25°C), keeping other aspects of the reaction buffer unchanged (Figure 5). *Tth* DNA ligase shows a ∼2.5-fold increase in initial velocity for the ligation of an isolated nick substrate when the buffer pH is raised from 7.5 to 8.0 (Supplementary Figure S5B), with no significant change when the pH was further increased to 8.5 or 9.0. This result matches the previously observed optimum pH range for this enzyme (19). The amount of observed mismatch ligation increased slightly when the pH is increased from 7.5 to 8.0, likely due simply to this ∼2.5-fold increase in activity of the enzyme, allowing a higher chance of mismatch products to be captured by ligation before melting and reannealing. In particular, increased upstream G:T and T:G mismatches were observed at higher pH, as well as slight increases in the yield of most observed downstream probe mismatch ligation products. At pH 8.5 and 9.0 the patterns look similar to ligation at pH 8.0. Interestingly, for a second ligase tested (9°N DNA ligase, see Supplementary Figure S6), increasing the pH increased fidelity substantially despite this ligase having a very similar activity/pH profile to *Tth* DNA ligase. Thus, the effects of buffer pH on fidelity appear to be more complex than simply increased activity resulting in increased yields of the mismatch products, and may need to be empirically tested for each enzyme.

### Effect of monovalent cations on mismatch ligation

It has been previously reported that addition of monovalent cations increases the fidelity of T4 DNA ligase and other ligases (8,9,26). In this study, the mismatch panel was used to screen the effects of systematically increasing the KCl concentration on *Tth* DNA ligase fidelity. Monovalent cation concentrations across the range of 50–200 mM were tested at pH 7.5 and 8.5. Previous reports have placed the ionic strength optimum at around 100 mM for *Tth* DNA ligase (19). Activity assays at 55°C (Supplementary Figure S5C) showed little effect on initial velocity of nick ligation at 25–150 mM KCl, with a drop to ∼ 50% activity at 200 mM and low activity at higher ionic strengths. This trend was the same for both buffer pHs tested (pH 7.5 and 8.5)

**Figure 6.**
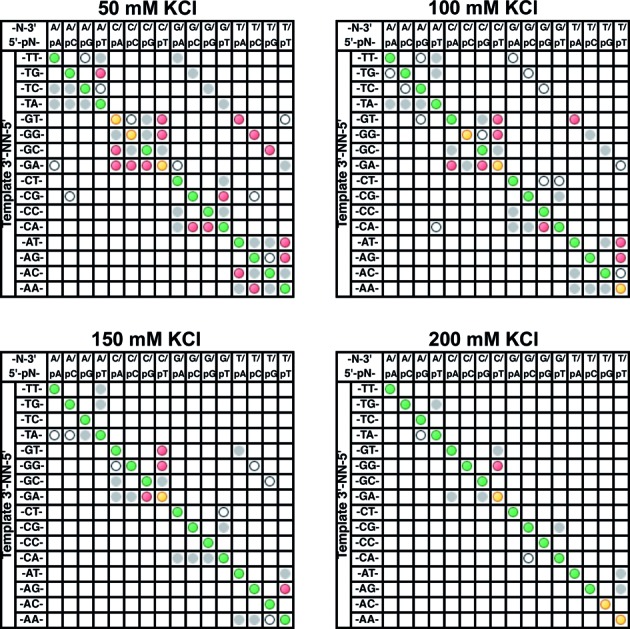
Mismatch ligation panel profiles for the reaction of the substrate panel with 2 nM *Tth* DNA ligase at buffer pH 8.5 with 50–200 mM KCl. Each reaction well contained 200 nM FAM-labeled oligonucleotides (see Materials and Methods for the composition of an individual substrate pool). Reactions were incubated for 30 min at 55°C in the standard reaction buffer at pH 8.5 with added KCl to achieve the desired final concentration. Note the standard buffer includes 25 mM KCl. The stated pH of the buffer stocks (Amresco) were determined at 25°C, not the reaction temperature. Products were analyzed via CE and the data are displayed as described in the Materials and Methods section ‘CE Data Processing and Analysis’ and the caption of Figure 3.

Figure 6 shows the effect of increasing salt concentration on the mismatch discrimination of *Tth* DNA ligase. An increase in KCl concentration steadily reduces the observed yields of mismatch ligation products, even in the concentration range where ligase activity on a fully WC base-paired nick substrate is unaffected. Significant improvements in fidelity are achieved at 100–200 mM KCl with high yields of the correctly WC base-paired substrate observed and greatly reduced yields of mismatch ligation. This result may seem counterintuitive due to the well-known ability of Na^+^ and K^+^ to stabilize the dsDNA double helix, increasing the *T*_m_ quite significantly over the concentrations used in this study (52). However, it has been shown that Mg^2+^ at concentrations typically used in nucleic acid enzyme reactions dominates the helix stabilization effect, and increasing salt in the presence of 10 mM MgCl_2_ is expected to only slightly affect the *T*_m_ (52). The increase in fidelity is most likely due to the monovalent cations affecting the binding of DNA by the ligase, weakening the interaction and consequently raising the *K*_m_. This effect appears to be stronger on mismatched substrates than on fully base-paired substrates, with the net result that the ratio of the catalytic efficiencies of the base-paired to mismatched substrates increases (9). Addition of KCl to standard reaction buffers thus appears to be a simple way to improve ligation discrimination, and adding the highest concentration of monovalent cations the ligase will tolerate without significant activity loss can improve fidelity substantially.

## CONCLUSION

A multiplexed substrate design combined with CE separation and detection of product oligonucleotides has allowed for a rapid high throughput screen for ligase fidelity. The current substrate allows for all 256 possible base combinations at a ligation junction to be screened in a single experiment, with up to six conditions in a single 96-well plate. The CE traces were acquired on a 96-capillary array, allowing many plates to be processed per day, and the data could be analyzed quantitatively using peak picking programs and standard analysis programs such as Excel. This method has allowed us to explore the ligation fidelity profile of *Tth* ligase in great depth, exploring the effects of temperature buffer pH, and monovalent cation concentration on the ligation of every mismatch and WC base-pair combination at once. From the parameters studied, it appears the best conditions for high fidelity and high activity are to use a buffer pH of ∼8.5 and a monovalent cation concentration of 150–200 mM Na^+^ or K^+^ ions, with a reaction temperature at or just above the *T*_m_ of the probe set used. Care must be taken to match the *T*_m_ of all probes used in one reaction to allow temperature optimization of fidelity.

The method here can be easily extended to examine additional buffers or enzymes, permitting the rapid evaluation of nucleic acid ligases and reaction conditions to develop next generation enzymes. Simple modifications to the substrate pool would permit this method to be used to screen for fidelity on other substrates, such as fully RNA or DNA/RNA hybrid structures. In principle, as long as all products are of unique lengths, the structures that can be explored are limited only by what can be synthesized. Indeed there is potential for even greater multiplexing by using additional fluorophores as well as the length of the oligonucleotides to distinguish starting materials and products. As can be seen by both this methodology and those examples described in the companion manuscript (46), CE provides an extremely flexible platform for the design of enzyme assays for the study of enzymes that act on nucleic acid substrates. The ease of product identification, quantitation, the ability to monitor the formation of multiple products and the ability to examine many reactions in parallel greatly speed generation and analysis of reaction data. These methods are invaluable tools for labs engaged in the study of nucleic acid enzymes.

## Supplementary Material

SUPPLEMENTARY DATA
