# Supplementary material for: A high-throughput assay for the comprehensive profiling of DNA ligase fidelity
Source: Nucleic Acids Res. 2015 Sep 13;44(2):e14. doi: 10.1093/nar/gkv898 (PMC4737175; doi:10.1093/nar/gkv898)
Supplement: SUPPLEMENTARY DATA [file supp_44_2_e14__index.html]

A high-throughput assay for the comprehensive profiling of DNA ligase fidelity — A high-throughput assay for the comprehensive profiling of DNA ligase fidelity — SUPPLEMENTARY DATA 

# A high-throughput assay for the comprehensive profiling of DNA ligase fidelity

## SUPPLEMENTARY DATA

- SUPPLEMENTARY DATA
