# Supplementary material for: A high-throughput assay for the comprehensive profiling of DNA ligase fidelity
Source: Nucleic Acids Res. 2015 Sep 13;44(2):e14. doi: 10.1093/nar/gkv898 (PMC4737175; doi:10.1093/nar/gkv898)

A high-throughput assay for the comprehensive profiling of DNA ligase fidelity

Gregory J. S. Lohman*, Robert J. Bauer, Laurie Mazzola, Joanna Bybee, Danielle Rivizzigno, Beth Ann Cantin and Thomas C. Evans, Jr.*

New England BioLabs, Inc., Ipswich, MA, 01938-2723, USA

*To whom correspondence should be addressed. Thomas C. Evans, Jr. Tel: 978-927-5054; Fax: 978-921-1350; Email: evanst@neb.com
Correspondence may also be addressed to: Gregory J. S. Lohman Tel: 978-998-7916; Fax: 978-921-1350; Email: ohman@neb.com

SUPPORTING INFORMATION

Figures S1 – S6

Figure S1. Sequences of oligonucleotides used in the assembly of the ligase fidelity panel described in the Materials and Methods, main text.

**Acceptor-A**	TAT AAC TTT ACT TCT ATT GA
**Acceptor-C**	CC AGG AGA TAT AAC TTT ACT TCT ATT GC
**Acceptor-G**	T CGT TTA GCC AGG AGA TAT AAC TTT ACT TCT ATT GG
**Acceptor-T**	CCT CTA CTT CGT TTA GCC AGG AGA TAT AAC TTT ACT TCT ATT GT
**Donor-pA**	pAGA TGG GAC CTA CAA TGT ACC AGA AGC GTC-FAM
**Donor-pC**	pCGA TGG GAC CTA CAA TGT ACC AGA AGC GTC TC-FAM
**Donor-pG**	pGGA TGG GAC CTA CAA TGT ACC AGA AGC GTC TCT C-FAM
**Donor-pT**	pTGA TGG GAC CTA CAA TGT ACC AGA AGC GTC TCT CTC-FAM
**Splint-TT**	GAC GCT TCT GGT ACA TTG TAG GTC CCA TCT TCA ATA GAA GTA AAG TTA TA
**Splint-GT**	GAC GCT TCT GGT ACA TTG TAG GTC CCA TCG TCA ATA GAA GTA AAG TTA TA
**Splint-CT**	GAC GCT TCT GGT ACA TTG TAG GTC CCA TCC TCA ATA GAA GTA AAG TTA TA
**Splint-AT**	GAC GCT TCT GGT ACA TTG TAG GTC CCA TCA TCA ATA GAA GTA AAG TTA TA
**Splint-TG**	GAC GCT TCT GGT ACA TTG TAG GTC CCA TCT GCA ATA GAA GTA AAG TTA TA
**Splint-GG**	GAC GCT TCT GGT ACA TTG TAG GTC CCA TCG GCA ATA GAA GTA AAG TTA TA
**Splint-CG**	GAC GCT TCT GGT ACA TTG TAG GTC CCA TCC GCA ATA GAA GTA AAG TTA TA
**Splint-AG**	GAC GCT TCT GGT ACA TTG TAG GTC CCA TCA GCA ATA GAA GTA AAG TTA TA
**Splint-TC**	GAC GCT TCT GGT ACA TTG TAG GTC CCA TCT CCA ATA GAA GTA AAG TTA TA
**Splint-GC**	GAC GCT TCT GGT ACA TTG TAG GTC CCA TCG CCA ATA GAA GTA AAG TTA TA
**Splint-CC**	GAC GCT TCT GGT ACA TTG TAG GTC CCA TCC CCA ATA GAA GTA AAG TTA TA
**Splint-AC**	GAC GCT TCT GGT ACA TTG TAG GTC CCA TCA CCA ATA GAA GTA AAG TTA TA
**Splint-TA**	GAC GCT TCT GGT ACA TTG TAG GTC CCA TCT ACA ATA GAA GTA AAG TTA TA
**Splint-GA**	GAC GCT TCT GGT ACA TTG TAG GTC CCA TCG ACA ATA GAA GTA AAG TTA TA
**Splint-CA**	GAC GCT TCT GGT ACA TTG TAG GTC CCA TCC ACA ATA GAA GTA AAG TTA TA
**Splint-AA**	GAC GCT TCT GGT ACA TTG TAG GTC CCA TCA ACA ATA GAA GTA AAG TTA TA

Figure S2. (A) CE trace of the donor mixture used for the preparation of panel substrate pools. The oligonucleotides were mixed based on the reported OD yields from IDT and eluted and analysed via CE. The sample trace shows four peaks: from left to right, the pA, pC, pG, and pT donors. Note that the FAM-labeled oligonucleotides run fast compared to the LIZ-labeled size standards, such that the 30, 32, 34, and 36 base oligonucleotides elute at ~11, 13.5, 17.5 and 19 bases relative to the size standard. The area of each peak was averaged over three runs and the molar ratio calculated to be 0.247 ± 0.004 : 0.260 ± 0.001 : 0.249 ± 0.003 : 0.244 ± 0.002 pA:pC:pG:pT. (B) HPLC elution of the mixture of the four acceptor nucleotides, detection by UV spectroscopy at 260 nM. As shown in the inset table, the peak area of each was normalized by the predicted extinction coefficient for each oligonucleotide. Peaks were identified by retention time compared to separate elution of each oligonucleotide. The molar ratio of the acceptor mix was found to be very close to 1:1:1:1 A:C:G:T (average of three runs).

A

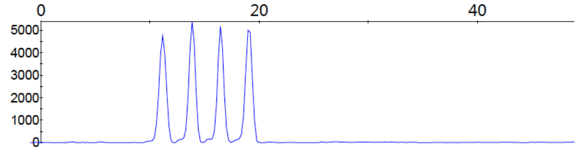

	pA	pC	pG	pT
**AVG**	0.247	0.26	0.249	0.244
**stdev**	0.004	0.001	0.003	0.002

B

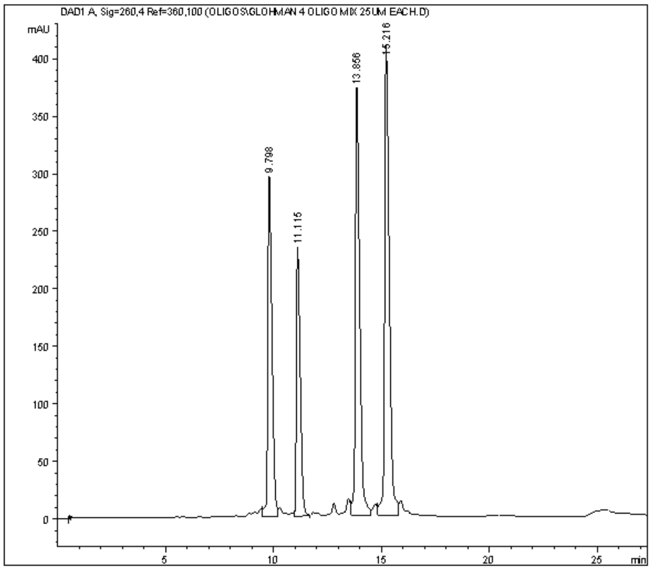

oligo	extinction coeff	Area%	area% / ex	Molar Ratio
A	193200	15.918	0.00008239	1.00
C	269400	21.937	0.00008143	0.99
G	347900	28.359	0.00008151	0.99
T	411700	33.786	0.00008206	1.00

Figure S3. Mismatch ligation panel profiles for the reaction of the standard substrate panel under conditions resulting in lower fidelity (increased mismatch ligation). (A) 20 nM Tth DNA ligase in standard reaction buffer at pH 7.5Reactions were incubated for 30 min at 55 °C. (B) 2 nM T4 DNA ligase in T4 DNA Ligase reaction buffer (50 mM Tris pH 7.5, 10 mM $MgCl_2$, 10 mM DTT, 1 mM ATP), incubated 30 min at 37 °C. Each reaction well contained 200 nM FAM-labeled oligonucleotides (see Materials and Methods for the composition of an individual substrate pool). At this reaction temperature, probes could not rapidly melt and reanneal and the maximum observed yield for any given peak was reduced as compared to reactions run at higher reaction temperature. Products were analyzed via CE and the data is displayed as described in the Materials and Methods section "CE Data Processing and Analysis" in the main text. Here we observed almost no discrimination against mismatches on the phosphorylated downstream probe side or against upstream T:T, T:G, G:T, C:A, or A:C mismatches, though upstream probe A:G and G:A mismatches were not ligated. T4 DNA ligase could even measurably ligate some double mismatches involving G:T, T:G and T:T mispairs on both sides of the ligation junction.

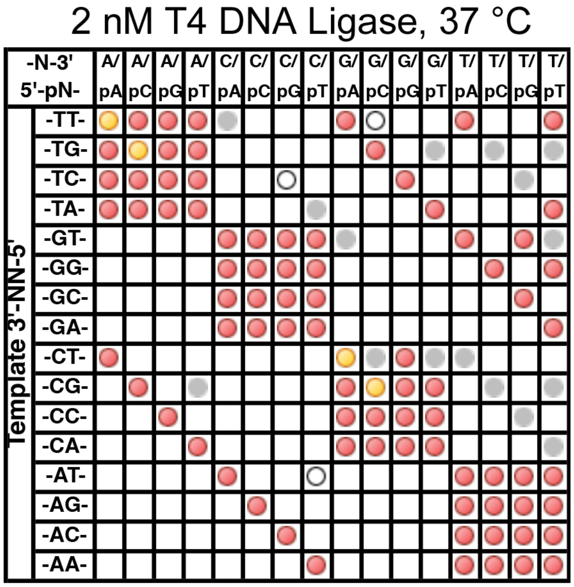

Figure S4. Mismatch ligation panel profiles for the reaction of an alternative substrate panel designed with a higher T_m (~75 °C for both acceptor and donor). Reaction conditions were 2 nM *Tth* DNA ligase in the standard reaction buffer at pH 7.5, incubated for 30 min at 65 °C or 75 °C. Products were analyzed via CE and the data is displayed as described in the Materials and Methods section "CE Data Processing and Analysis" from the main text.

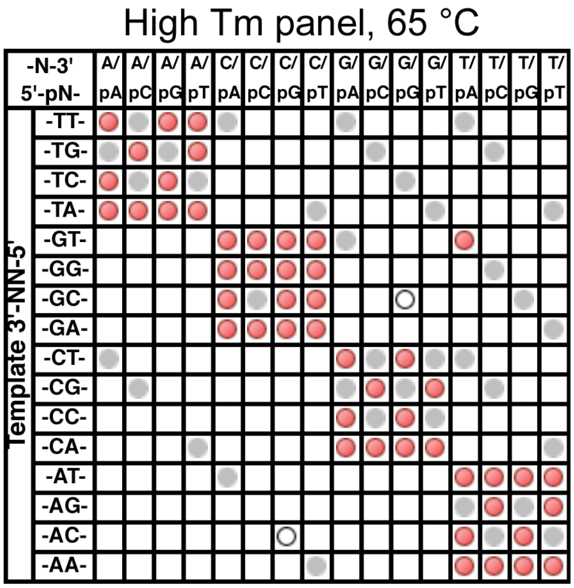

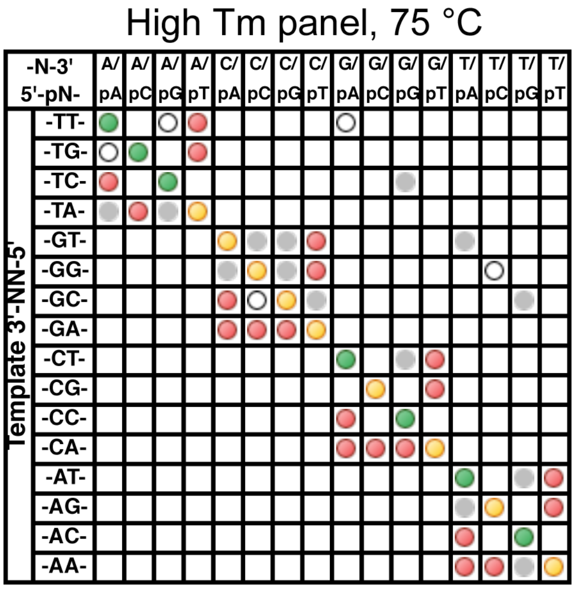

Figure S5. Activity of *Tth* DNA ligase as determined by the initial velocity assay described in detail in the Materials and Methods, main text. Briefly, 0.25 nM *Tth* DNA ligase was incubated with 100 nM nicked substrate (T_m ~ 75 °C) in (A) standard ligation buffer, pH 7.5, at 45, 55, 65 or 75 °C; (B) ligation buffer at pH 7.0, 7.5, 8.0, 8.5, or 9.0 at 55 °C; or (C) standard buffer at pH 7.5 or pH 8.5 at 55 °C with KCl added to a final concentration of 25, 50, 100, 150, 200, 250, or 300 mM. The rates were determined by linear fits to the first 5-20% of reaction and are the calculated slopes/[*Tth* Ligase] in units min^{-1} (average of at least three experiments, error bars are one standard deviation from the mean).

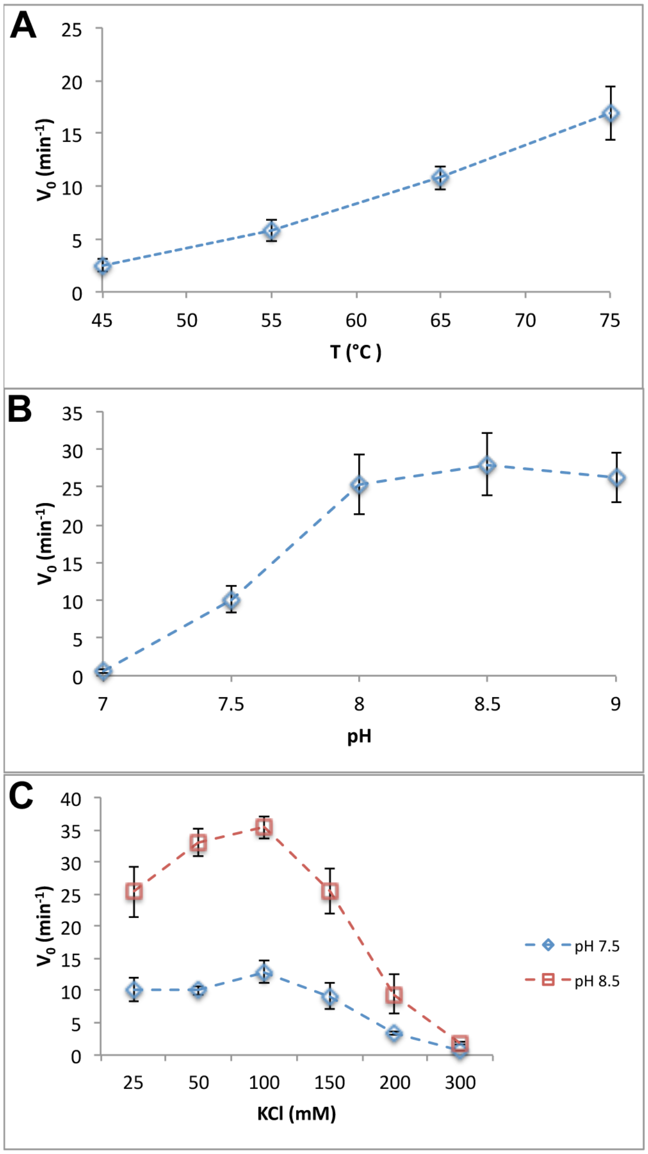

Figure S6. Mismatch ligation panel profiles for the reaction of the standard substrate panel with 20 nM 9°N DNA ligase at buffer pH 7.5 – 9.0. Each reaction well contained 200 nM FAM-labeled oligonucleotides (see Materials and Methods for the composition of an individual substrate pool). Reactions were incubated for 30 min at 55 °C in the 9°N reaction buffer (10 mM Tris-HCl, 600 µM ATP, 2.5 mM $MgCl_2$, 2.5 mM DTT, 0.1 % Triton X-100) at pH 7.5, 8.0, 8.5, or 9.0. The stated pH of the buffer stocks (Amresco) was determined at 25 °C, not the reaction temperature. Products were analyzed via CE and the data is displayed as described in the Materials and Methods section “CE Data Processing and Analysis” in the main text.

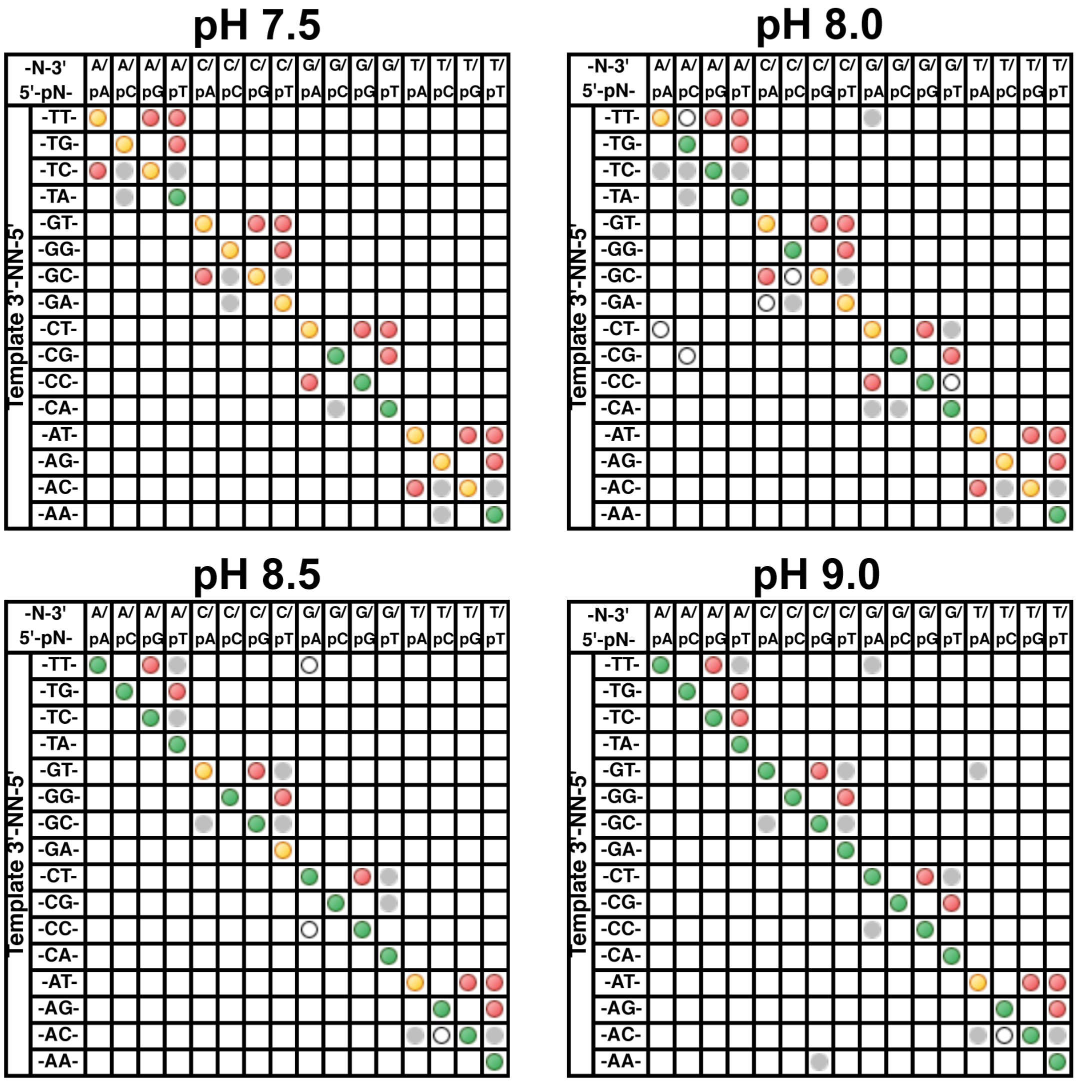

Supplement: SUPPLEMENTARY DATA [file supp_gkv898_nar-01316-met-k-2015-File009.pdf]
